# A Cross-Sectional Study of Breast Cancer Surgery and the Cost Based on Data From 77 Chinese 3A Hospitals in 2015

**DOI:** 10.3389/fonc.2022.857359

**Published:** 2022-04-26

**Authors:** Rong Zhao, Meng Jin, Jinnan Gao, Linhui Zhang, Liyuan Tao, Xiaoyuan Bao

**Affiliations:** ^1^ Department of Breast Surgery, Shanxi Bethune Hospital, Shanxi Academy of Medical Sciences, Tongji Shanxi Hospital, Third Hospital of Shanxi Medical University, Taiyuan, China; ^2^ Research Center of Clinical Epidemiology, Peking University Third Hospital, Beijing, China; ^3^ Institute of Rehabilitation Industry, Fujian University of Traditional Chinese Medicine, Fuzhou, China; ^4^ Peking University Medical Informatics Center, Peking University, Beijing, China

**Keywords:** breast neoplasms, China, cross-sectional studies, cost of illness, real-world study, surgery

## Abstract

**Purposes:**

We aimed to clarify the real-world status of breast cancer surgery and the cost in China.

**Methods:**

This cross-sectional survey relied on data obtained from the hospitalization summary reports (HSRs) in 77 top-ranked (grade 3A) hospitals in China to analyze breast cancer patients who underwent surgery between January 2015 and December 2015. The surgery and cost were mainly evaluated.

**Results:**

Overall, 31,900 breast cancer patients underwent surgeries in 77 hospitals. The mean age in our study was 51.5 years (SD, 11.7 years). The primary types of surgical procedures were mastectomy (n = 24,629, 77.2%) and breast-conserving surgery (6,210, 19.5%). The rate of mastectomy was the highest at age band 50–65 years (n = 10,861, 82.1%) and in non-first-tier cities (n = 7,651, 88.4%) as well as in Northeast China (n = 3,107, 93.2%). The rate of breast-conserving surgery was less than 10% in non-first-tier cities (9.8%), Southwest China (6.1%), and Northeast China (5.8%). The median cost was $3,352.4 (interquartile range (IQR), $2,492.6–4,588.0). Mastectomy cost was significantly higher than breast-conserving surgery cost in both different city tiers and regional distribution except Northeast China (p < 0.001).

**Conclusions:**

This study demonstrated that the main breast cancer surgery in Chinese 3A hospitals was mastectomy and that the cost varied both across and within geographic regions and city tiers. This information helps describe the real-world status of breast surgery and the cost in China.

## Introduction

Breast cancer is the most common female cancer all over the world (1). Although the incidence and mortality rates of breast cancer in China are relatively low, they are both on the rise. Due to the large population base, the incidence and death of breast cancer in China rank the first in the world (2, 3).

Compared with other countries, for example, the United States, in which the median diagnosed age of breast cancer is more than 60 years (4), in China, breast cancer occurs at a younger age in women, with the median age at diagnosis of 48–50 years (5–7). Breast cancer in China is with more frequent advanced-stage presentation (6), and there are regional variations in incidence (8). The average total treatment cost for each new case in China is the lowest in the world, but as the largest low-income or middle-income country, breast cancer carries a heavy burden on Chinese patients (9). Another characteristic of Chinese women is their smaller breast size (10). All of these would affect Chinese patients’ and surgeons’ choice of treatment.

Surgery is one of the main methods in the treatment of breast cancer and influences patients’ quality of life. Breast-conserving surgery (BCS) is a standard treatment of early-stage breast carcinoma (11), while findings from a Chinese nationwide survey from 1999 to 2008 showed that mastectomy continues to be the main operation, accounting for 88.8% for primary breast cancer. By contrast, the incidence of mastectomy was 36% in the United States (12). In addition to mastectomy and BCS, oncoplastic surgery could be an option (13, 14), which has developed at a rapid pace and is being performed all over the world (15). However, the oncoplastic rate in China remains low, and most hospitals do not possess this type of surgery.

Most of the aforementioned data were published before 2010 and are therefore relatively old, so it is necessary to review the status quo of surgical treatment of breast cancer in China. Using data from a survey in 2015, we aimed to clarify the current real-world breast surgery in a Chinese population and to provide a reference for the formulation of strategies.

## Methods

### Data Source

Data were obtained from the hospitalization summary reports (HSRs) in 77 top-ranked (grade 3A) hospitals in China from January 2015 to December 2015. Hospitals in China are classified into three grades. Grade 3A hospitals are with the highest rank.

The HSRs are electronically submitted to the Beijing Municipal Health Bureau, containing information on hospitals, demographics, dates of admission and discharge, diagnoses (pre- and post-hospitalization), treatments, and hospitalization costs. Post-hospitalization diagnoses were coded by the International Classification of Diseases, 10th Revision (ICD-10) codes, and surgical operations were coded by International Classification of Diseases, 9th Revision, Clinical Modification (ICD-9-CM) codes.

This article was considered exempt from ethics review because it used data collected for administrative purposes without any personal identifiers. Patients who underwent surgery for primary breast malignancy were identified.

### Study Population

In the present study, patients with breast cancer were identified from the database using the ICD-10 codes C50 (the first listed diagnosis). Then 85.2000x001, 85.2000x002, 85.3600x001, 85.8100, 85.9x200x001, and 85.9200 items were deleted, as those codes represent surgical management of surgical complications. For example, 85.2000x001 represents “Breast skin and subcutaneous necrotic tissue excision and debridement.” Patients who had undergone surgery were identified using the ICD-9-CM codes 85.2–85.9. To avoid the omission, all surgery and disposal items were contacted, and the Chinese terms of “surgery” were applied to identify additional eligible patients. The main surgery was identified in a logical way. For example, if a patient’s surgery codes included “mastectomy” and “BCS,” the main code should be “mastectomy” rather than “BCS,” as she should not be qualified for BCS and should undergo mastectomy immediately.

### Statistical Analysis

All data were analyzed through Statistical Package for the Social Sciences (SPSS) V.26. Ages were categorized into four groups: <35, 35–49, 50–65, and >65. Hospitals were categorized into 7 regions (North China, South China, Southwest China, Northeast China, East China, Central China, and Northwest China) according to geographical distribution and 2 city tiers (first tier and non-first tier, according to official list). Categorical variables are reported as a proportion (%), and numerical data are reported as mean ± SD or median and corresponding 25th and 75th percentiles (interquartile range (IQR)). ANOVA, independent t-test, Mann–Whitney U test, and chi-squared (χ^2^) test were performed as appropriate for statistical analysis. All p-values were considered statistically significant at two-tailed p < 0.05.

## Results

### Characteristics of Patients

A total of 31,900 patients who underwent breast cancer surgery were identified from the database. Demographics and pathologic characteristics, surgery, cost, city level, and regions are summarized in **Table 1**.

**Table 1:** Characteristics of patients.

	N	%
Age (years)		
Mean (SD)	51.5 (11.7)	
<35	1,962	6.2
35–49	12,928	40.5
50–65	13,221	41.4
>65	3,789	11.9
Surgical method		
Mastectomy	24,629	77.2
BCS	6,210	19.5
Reconstruction	787	3.7
Autogenous reconstruction	138	0.8
Pedicled flaps	123	0.4
LADO	104	0.3
TRAM	19	0.1
Free flaps	15	
Implant	561	1.8
Other reconstruction	88	0.3
Palliative surgery	16	0.1
Others	258	0.8
Male breast cancer	26	0.1
City tiers		
First-tier cities	23,241	72.9
Non-first-tier cities	8,659	27.1
Regions		
North China	7,225	22.6
South China	2,439	7.6
Southwest China	1,786	5.6
Northeast China	3,334	10.5
East China	11,022	34.6
Central China	4,095	12.8
Northwest China	1,999	6.3
Pathological type	7,668	100
Invasive carcinomas	7,381	96.3
Invasive ductal carcinomas	5,694	74.3
Invasive lobular carcinoma	216	2.8
Mucinous carcinoma	100	1.3
Other types	1,371	17.9
Carcinoma *in situ*	268	3.5
DCIS	174	2.3
Paget disease	94	1.2
Other types	19	0.2
The total cost ($)		
Median (IQR)	3,352.4 (2,492.6–4,588.0)	
Traditional Chinese medicine($)		
The number of users	8,184	25.7
Median (IQR)	90.9 (26.6–199.0)	

BCS breast-conserving surgery; LADO, lattisimus dorsi flap; TRAM, transverse rectus abdominis myocutaneous flap; DCIS, ductal carcinoma in situ; IQR, interquartile range.

The mean age in our study was 51.5 years (SD, 11.7 years). Most patients were in the 35–65 age range (81.9%), and 46.7% of the patients were diagnosed before 50 years of age. The primary types of surgical procedure were mastectomy and BCS. Nearly four-fifths of the patients (n = 24,629, 77.2%) underwent mastectomy, and less than one-fifth (n = 6,210, 19.5%) underwent BCS. The rate of reconstruction was low (n = 787, 3.7%), and the main reconstruction surgery was implant (n = 561, 1.8%). In our study, 26 patients (0.1%) was male. Of the patients, 72.9% (n = 23,241) were from first-tier cities (first-tier cities cover 19 cities), and more than half of the patients came from East (n = 11,022, 34.6%) or North China (n = 7,225, 22.6%). There were less patients from Southwest (n = 1,786, 5.6%), Northwest (n = 1,999, 6.3%), and South China (n = 2,439, 7.6%).

Only 7,668 patients had available pathological information. Invasive cancer accounted for 96.3% (n = 7,381), and carcinoma *in situ* accounted for 3.5% (n = 268). The median cost was $3,352.4 (IQR, $2,492.6–4,588.0). More than a quarter of the patients (n = 8,184, 25.7%) used traditional Chinese medicine (TCM) during the hospital stay, and the median cost was $90.9 (IQR, $26.6–199.0).

### Age Distribution of City Tiers and Regions

Patients in first-tier cities tended to be younger than in non-first-tier cities (mean (SD), 50.9 years (11.2 years) vs. 51.8 years (11.8 years), p < 0.001). Patients younger than 50 years were 45.7% in first-tier cities and 49.1% in non-first-cities.

Age distributions were different among regions (p < 0.001). The oldest populations were located in North China [mean (SD), 53.1 years (12.1 years)] (**Table 2**).

**Table 2:** Age (years) distribution of city tiers and regions.

	Mean (SD)	<35	35–49	50–65	>65	p
City tiers						<0.001
First-tier cities	51.8 (11.8)	1,472 (6.3)	9,167 (39.4)	9,678 (41.6)	2,924 (12.6)	
Non-first-tier cities	50.9 (11.2)	490 (5.7)	3,761 (43.4)	3,543 (40.9)	865 (10.0)	
Regions						<0.001
North China	53.1 (12.1)	400 (5.5)	2,565 (35.5)	3,159 (43.7)	1,101 (15.2)	
South China	49.3 (11.4)	219 (9.0)	1,109 (45.5)	901 (36.9)	210 (8.6)	
Southwest China	50.2 (10.9)	88 (4.9)	888 (49.7)	647 (36.2)	163 (9.1)	
Northeast China	51.7 (11.0)	164 (4.9)	1,309 (39.3)	1,504 (45.1)	357 (10.7)	
East China	52.1 (12.0)	692 (6.3)	4,225 (38.3)	4,672 (42.4)	1,433 (13.0)	
Central China	49.3 (10.5)	273 (6.7)	2,003 (48.9)	1,540 (37.6)	279 (6.8)	
Northwest China	51.4 (11.8)	126 (6.3)	829 (41.5)	798 (39.9)	246 (12.3)	

### Breast Surgery and the Cost

There were significant age, city tiers, regions, and cost differences across surgical groups (p < 0.001). The mean age of the patients who underwent reconstruction was younger than 50 years (lattisimus dorsi flap, 43.5 years (SD, 9.4 years); transverse rectus abdominis myocutaneous flap, 45.6 years (SD, 9.1 years); free flaps, 46.7 years (SD, 12.0 years); implant, 40.1 years (SD, 8.8 years); other reconstruction, 45.4 years (SD, 9.6 years)). The rate of mastectomy was the highest at age band 50–65 (n = 10,861, 82.1%), and the rate of BCS was the highest at age band <35 (n = 551, 28.1%).

The rate of BCS was 23.1% in first-tier cities; however, it was less than 10% (9.8%) in non-first-tier cities. In terms of geographic division, the highest rate of mastectomy was in Northeast China (93.2%, n = 3,107), ranking no. 1, and the lowest rate of mastectomy was in South China (69.4%, n = 1,692) and North China (69.6%, n = 5,026). Reconstruction rates were low in all regions. Reconstruction expended more, and the first was free flap (median, $7,185.5; IQR, $4,083.3–8,601.3) (**Table 3**).

**Table 3:** Breast surgery distribution of age, city tiers, regions, and the total cost.

	Mastectomy	BCS	Reconstruction					Palliative surgery	Others	p
			Autogenous reconstruction			Implant	Other reconstruction			
			Pedicled flaps		Free flaps					
			LADO	TRAM						
Age (years)										<0.001
Mean (SD)	52.2 (11.3)	50.2 (12.7)	43.5 (9.4)	45.6 (9.1)	46.7 (12.0)	40.1 (8.8)	45.4 (9.6)	57.8 (14.4)	50.1 (11.1)	
<35	1,191 (60.7)	551 (28.1)	17	3	3	166	9	0	22	<0.001
35–49	9,587 (74.2)	2,782 (21.5)	61	10	5	320	54	5	104	
50–65	10,861 (82.1)	2,112 (16.0)	25	6	6	71	22	8	110	
>65	2,990 (78.9)	765 (20.2)	1	0	1	4	3	3	22	
City tiers										<0.001
First-tier cities	16,978 (73.1)	5,363 (23.1)	79	17	13	492	59	15	225	
Non-first-tier cities	7,651 (88.4)	847 (9.8)	25	2	2	69	29	1	33	
Regions										<0.001
North China	5,026 (69.6)	1,969 (27.3)	17	1	4	122	11	0	75	
South China	1,692 (69.4)	668 (27.4)	7	6	1	54	3	0	8	
Southwest China	1,596 (89.4)	109 (6.1)	7	0	0	53	19	0	2	
Northeast China	3,107 (93.2)	195 (5.8)	1	1	0	19	6	0	5	
East China	8,306 (75.4)	2,187 (19.8)	62	11	9	261	43	15	128	
Central China	3,181 (77.7)	848 (20.7)	1	0	1	34	1	1	28	
Northwest China	1,721 (86.1)	234 (11.7)	9	0	0	18	5	0	12	
The total cost ($)										<0.001
Median (IQR)	3,454.6 (2,601.9–4,682.5)	2,775.4 (1,953.2–3,995.1)	4,064.3 (3,389.2–5,299.3)	4,377.4 (3,785.4–6,943.3)	7,185.5 (4,083.3–8,601.3)	4,411.5 (3,091.8–6,632.6)	4,199.6 (3,366.8–5,113.5)	1,992.7 (1,185.7–2,431.5)	2,139.0 (1,428.7–3,621.1)	

BCS, breast-conserving surgery; LADO, lattisimus dorsi flaps; TRAM, transverse rectus abdominis myocutaneous flaps.

**Figure 1:**
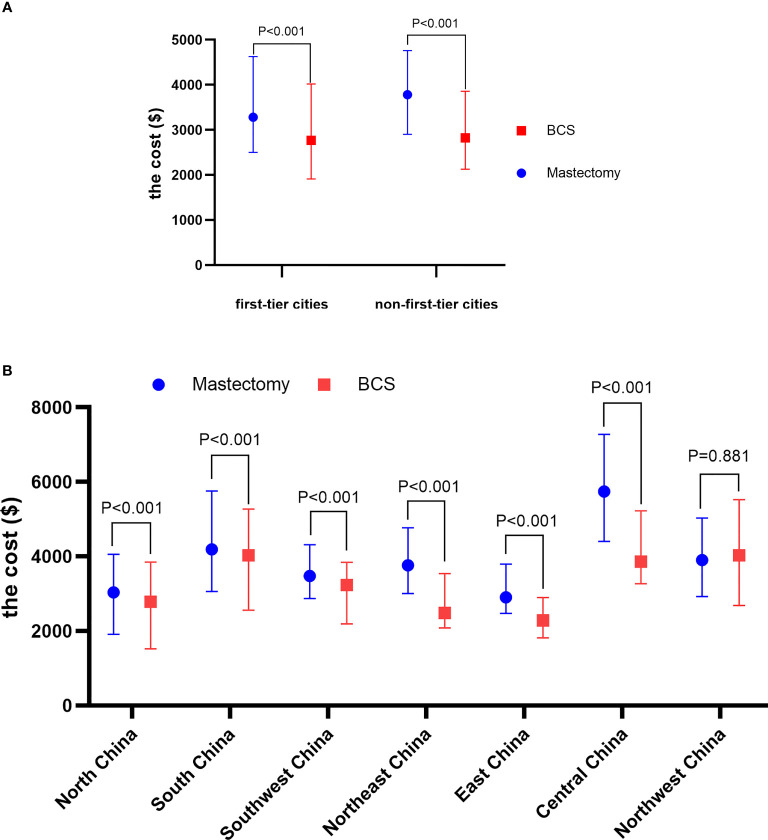
The median cost of mastectomy and BCS of different city tiers **(A)** and regions **(B)**. BCS, breast-conserving surgery.

In addition, mastectomy cost was significantly higher than BCS cost in both different city tiers and regional distribution (p < 0.001) except in Northeast China (p = 0.881) (**Figure 1** and **Supplementary Table 1**).

## Discussion

Although our study only included part of Chinese breast cancer patients who underwent surgery from grade 3A hospitals, accounting for about one-tenth of the total incidence in 2015 (16), it is still the largest study to reflect the latest surgery status of breast cancer in China. The age characteristics of the newly diagnosed cases in our study are consistent with previous reports (5–7). Younger age and higher density are a great challenge for the Chinese government in developing screening strategies, as the benefit of mammography remains controversial in women younger than 50 years (17), and half of the Chinese patients are within this range. However, the best recommendations of the age range for mammography screening are based on European and US trials, and China lacks large analogous trials. It is necessary to carry out corresponding research to better guide our clinical practice.

Compared with the United States (less than 40%) (12, 18), in China, mastectomy accounts for 77.2% of surgery for primary breast cancer in our study. South China and North China showed a low rate of mastectomy across the country. It may be due to these two regions containing hospitals from Beijing and Shanghai, which represent higher Chinese medical levels. It is consistent with the rate in first-tier cities. Notably, the rate in South China and North China was high at 70%, but our study only contains large 3A hospitals, so the real results might be worse. There are five possible reasons.

The first reason may be the high percentage of advanced stage at presentation. A previous study (6) revealed that Chinese patients were diagnosed 60.6% at stage I/II and 21.1% at stage III/IV disease, but disease presentations at stage III/IV (visit to oncologist firstly) might be substantially underestimated because most data on the stage at diagnosis are mainly collected from surgical departments. A business survey found that nearly two-thirds of Chinese breast cancer patients were diagnosed with advanced disease (9).

The second influence factor is small breast size. BCS is not suitable when the tumor–breast size ratio exceeds a certain value, which is common in China (6, 19). The third reason is that the development of breast reconstruction in China is less than ideal. Breast reconstruction can help patients achieve disease control and aesthetic appearance. However, the reconstruction rate in China is low, and only several hospitals meet the surgical qualification. A 2019 Chinese study (20) showed that the rate of implant-based breast reconstruction in all the patients who received mastectomy was only 7.06% (4,296/60,877).

The fourth reason is Chinese patients’ misunderstanding of breast surgery. Most of the patients have doubts about “less is more” and tend to regard breast conservation as disease residual. At the same time, they consider reconstruction as major surgery, which means complications, and they fear surgical complications (21). Patient education may help change this situation.

The average total treatment cost of a new case of breast cancer in China ranks low as compared with other countries (9). However, breast cancer is one of the leading causes of catastrophic medical expenditure in China and can rapidly impoverish families (9, 22). The cost of radiotherapy after breast conservation is a heavy burden (23). This may be the fifth reason for the low rate of breast conservation. However, the psychological effects of mastectomy could be higher than the cost of radiotherapy, and this problem could be managed with insurance facilities. Additionally, there is a wide range of variation both across and within geographic regions in the treatment and nursing cost, and it is hard to discern whether higher fees mean better service or survival. With the advancement of the diagnosis-related group (DRG) system in China (24), the gap in breast cancer treatment costs between and within regions would be greatly decreased. Also, based on our data, the median cost of BCS ($2,775.4) is lower than that of mastectomy ($3,454.6). In other words, BCS means more profit for surgeons. Maybe the implementation of the DRG system would encourage surgeons to promote breast-conserving surgery.

About 25% of breast cancer patients in our study have used TCM. The widespread use of traditional medicine is related to beliefs that it can alleviate toxic effects, promote recovery, and improve immune function and quality of life (25). It is different from modern medicine, based on the holistic approach and the Chinese materia medica to diagnose and treat the disease. TCM is an empirical science based on over 2,000 years of accumulated knowledge and practice (26). TCM is usually regarded as a complementary or “alternative” treatment of modern Western medicine in other countries (27). It is regarded the same in most hospitals in China except TCM hospitals. The limitation of TCM applications is mainly due to its lack of evidence-based features (28). The integration of scientific principles and techniques with traditional medicine will serve modern medicine well (29).

Our study has important limitations. Firstly, some patients received both surgery and chemotherapy during the same hospital stay, leading to the cost being not comparable to some extent. On the other hand, the cost in our study is the total cost, including medical insurance reimbursement, rather than patients’ own expense. So the cost in our study cannot reflect the personal medical expenditure. Secondly, our study lacks breast cancer stage; some of our inferences (for example, “this may be due to advanced stage”) are based on other studies rather than our data. More importantly, although we figured out that the data of breast cancer stage at present may be imprecise in China, we are unable to provide relevant data. Thirdly, the hospitals in our study are all at the 3A level, representing a high medical level in China. The data we present could not reflect the reality of breast cancer surgery and the cost. Fourthly, HSR data are still subject to some incomplete or inaccurately recorded information, for example, wrong ICD coding order in the medical records room. However, we checked the logical order, and errors in the data may have been reduced. Finally, the data we provided were a bit outdated (2015); however, they were the most current and large-scale datasets to date.

## Conclusion

Our study found that the main breast cancer operation in China in 2015 is mastectomy and that there is a wide cost range of variation both across and within geographic regions and city tiers. There are gaps in breast cancer surgery treatment in comparison to other countries. The Chinese government needs to take measures to improve the quality of treatment for breast cancer patients in China.

## Data Availability Statement

The raw data supporting the conclusions of this article will be made available by the authors, without undue reservation.

## Author Contributions

Conceptualization: JG, LT, RZ, and LZ. Project administration: LT. Methodology: LT and XB. Data curation: RZ and MJ. Formal analysis: LT. Manuscript preparation: JG and RZ. Final approval of manuscript: all authors.

## Funding

This work was supported by the National Natural Science Foundation of China [grant number: 82004210] and the National Key R&D Program of China (2018YFC1704400).

## Conflict of Interest

The authors declare that the research was conducted in the absence of any commercial or financial relationships that could be construed as a potential conflict of interest.

## Publisher’s Note

All claims expressed in this article are solely those of the authors and do not necessarily represent those of their affiliated organizations, or those of the publisher, the editors and the reviewers. Any product that may be evaluated in this article, or claim that may be made by its manufacturer, is not guaranteed or endorsed by the publisher.
