# Supplementary material for: A Cross-Sectional Study of Breast Cancer Surgery and the Cost Based on Data From 77 Chinese 3A Hospitals in 2015
Source: Front Oncol. 2022 Apr 26;12:857359. doi: 10.3389/fonc.2022.857359 (PMC9086896; doi:10.3389/fonc.2022.857359)
Supplement: Supplementary file 1 [file Table_1.docx]

Supplementary Material

# Supplementary Table

Supplement Table: The median (IQR) cost ($) of mastectomy and breast-conserving surgery of different city tiers and regions.

|  | Mastectomy | BCS | P |
| --- | --- | --- | --- |
| City tiers |  |  |  |
| First-tier cities | 3281.7(2502.6-4626.2) | 2769.3 (1910.6-4019.4 ) | <0.001 |
| Non-first-tier cities | 3783.0(2900.4-4761.8) | 2824.6 (2126.7-3855.1 ) | <0.001 |
| Regions |  |  |  |
| North China | 3036.2(1909.6-4056.6) | 2783.2 (1522.1-3849.2 ) | <0.001 |
| South China | 4192.2(3060.6-5754.2) | 4031.0 (2557.3-5267.9 ) | <0.001 |
| Southwest China | 3474.2(2871.2-4315.7) | 3234.6 (2190.1-3845.9 ) | <0.001 |
| Northeast China | 3763.0(3006.6-4769.3) | 2481.4 (2083.7-3543.7 ) | <0.001 |
| East China | 2902.4(2467.7-3797.4) | 2281.6 (1816.8-2899.9 ) | <0.001 |
| Central China | 5743.9(4405.0-7276.0) | 3860.8 (3271.4-5223.0 ) | <0.001 |
| Northwest China | 3901.7(2924.1-5028.0) | 4031.9 (2681.0-5522.7 ) | P=0.881 |

Abbreviations: BCS breast-conserving surgery.
